# *DRD2/CHRNA5* Interaction on Prefrontal Biology and Physiology during Working Memory

**DOI:** 10.1371/journal.pone.0095997

**Published:** 2014-05-12

**Authors:** Annabella Di Giorgio, Ryan M. Smith, Leonardo Fazio, Enrico D'Ambrosio, Barbara Gelao, Aldo Tomasicchio, Pierluigi Selvaggi, Paolo Taurisano, Tiziana Quarto, Rita Masellis, Antonio Rampino, Grazia Caforio, Teresa Popolizio, Giuseppe Blasi, Wolfgang Sadee, Alessandro Bertolino

**Affiliations:** 1 IRCCSS “Casa Sollievo della Sofferenza”, San Giovanni Rotondo, Italy; 2 Department of Pharmacology, Center for Pharmacogenomics, The Ohio State University, Columbus, Ohio, United States of America; 3 Group of Psychiatric Neuroscience, Department of Basic Medical Science, Neuroscience and Sense Organs, Aldo Moro University, Bari, Italy; 4 Cognitive Brain Research Unit, Department of Behavioral Sciences, University of Helsinki, Helsinki, Finland; 5 pRED, NORD DTA, F. Hoffman-La Roche Ltd., Basel, Switzerland; University of Texas at Dallas, United States of America

## Abstract

**Background:**

Prefrontal behavior and activity in humans are heritable. Studies in animals demonstrate an interaction between dopamine D2 receptors and nicotinic acetylcholine receptors on prefrontal behavior but evidence in humans is weak. Therefore, we hypothesize that genetic variation regulating dopamine D2 and nicotinic acetylcholine receptor signaling impact prefrontal cortex activity and related cognition. To test this hypothesis in humans, we explored the interaction between functional genetic variants in the D2 receptor gene (*DRD2*, rs1076560) and in the nicotinic receptor α5 gene (*CHRNA5*, rs16969968) on both dorsolateral prefrontal cortex mediated behavior and physiology during working memory and on prefrontal gray matter volume.

**Methods:**

A large sample of healthy subjects was compared for genotypic differences for *DRD2* rs1076560 (G>T) and *CHNRA5* rs16969968 (G>A) on prefrontal phenotypes, including cognitive performance at the N-Back task, prefrontal physiology with BOLD fMRI during performance of the 2-Back working memory task, and prefrontal morphometry with structural MRI.

**Results:**

We found that *DRD2* rs1076560 and *CHNRA5* rs16969968 interact to modulate cognitive function, prefrontal physiology during working memory, and prefrontal gray matter volume. More specifically, *CHRNA5*-AA/*DRD2*-GT subjects had greater behavioral performance, more efficient prefrontal cortex activity at 2Back working memory task, and greater prefrontal gray matter volume than the other genotype groups.

**Conclusions:**

The present data extend previous studies in animals and enhance our understanding of dopamine and acetylcholine signaling in the human prefrontal cortex, demonstrating interactions elicited by working memory that are modulated by genetic variants in *DRD2* and *CHRNA5*.

## Introduction

Working memory is a highly heritable complex cognitive trait [1]–[3], defined as the ability to keep information immediately available for a short period of time to solve a task that may be delayed; it is, therefore, a fundamental component of higher-level functions [4]. The prefrontal cortex has been identified as a key neocortical region supporting working memory [4], [5]. Previous functional imaging studies in humans have demonstrated that working memory prefrontal activity is also heritable [6], [7], suggesting the importance of prefrontal cortex function in generating and testing various neuroimaging intermediate phenotypes for complex genetic brain disorders.

Several neurotransmitters [8] and related genetic variation [9]–[11] modulate the physiology of prefrontal cortex and interact in determining neuronal response to cognitive stimuli. It is well known that dopamine critically modulates prefrontal neuronal *signal-to-noise* during working memory processes [12]. By differentially acting on dopamine D1 and D2 receptors, dopamine directly regulates firing of pyramidal neurons and of their GABA inhibitory surround within prefrontal cortex to focus prefrontal cortical resources to the task at hand [13], [14]. Recent studies in animal models also implicate a specific role for prefrontal acetylcholine [15]–[17]. For example, rhesus monkeys with selective lesions of cholinergic input from the basal forebrain to the lateral and orbital prefrontal cortex are unimpaired in tests of decision making and episodic memory that also require intact prefrontal cortex, but are severely impaired on a spatial working memory task [16]. Pharmacological studies in animals and in humans complement cholinergic lesion studies, specifically implicating nicotinic acetylcholine receptors (nAChRs) in working memory performance [18], [19]. Systemic administration of high doses of mecamylamine, a nicotinic receptor antagonist, as well as intracranial infusion of the antagonist dihydro-β-erythroidine in the frontal cortex of rats lead to significant working memory performance deficits in the radial arm maze [20], [21]. Conversely, agonist-mediated activation of AchRs improves working memory performance in rats [20], [22], rabbits [23], non-human primates [24], and abstinent smokers [25]. Moreover, transdermally-administered nicotine in humans also improves performance in a variety of recall tasks through non-selective stimulationof nAChRs [26].

Neuronal nAChRs are pentameric ligand-gated channels, distinguished on the basis of subunit stoichiometry (α2- α10, β2–β4) [27]. α4β2-containing receptors are present on multiple cell types in multiple layers (L) of the human prefrontal cortex [28], where they modulate layer-specific activity of pyramidal neurons [29]. Specifically, LII/III pyramidal neurons are inhibited by nAChR stimulation, while LV and LVI pyramidal neurons are prominently activated. α4β2 nAChRs incorporating the α5 accessory subunit (α4β2α5) are important players in the regulation of prefrontal neuronal plasticity [30], [31]. The α5 subunit is more densely expressed on soma and axons of pyramidal neurons LVI of the murine medial prefrontal cortex [29], [32]–[35]. This subunit substantially increases the conductance [31] and currents [36] of α4β2-containing nAChRs, and drives developmental changes in the morphology and activation of medial prefrontal cortex LVI pyramidal neurons [37].

Because both dopaminergic and cholinergic systems modulate pyramidal neuron firing in the prefrontal cortex, they likely interact to shape prefrontal neuronal plasticity critical for information processing. Pharmacological studies in rodents support a potential interaction between these two neuromodulators. Radial maze performance, a behavioral measure of working memory in rats, is improved after application of a nAChR agonist [38] but impaired by an antagonist [39]. Interestingly, the detrimental effect of the nAChR antagonist can be reversed by a dopamine D2 receptor agonist [40], while co-administration of a D2 receptor and of a nAChR antagonist leads to an even stronger impairment compared with the effect of each pharmacological challenge alone [41]. The interaction is specific for dopamine D2 receptors as dopamine D1 agonists do not neutralize the detrimental effect of nAChR antagonists [42]. However, studies have yet to be performed in humans to evaluate this potential interaction in terms of prefrontal physiology during executive and cognitive control processes.

In the present study, we evaluated this interaction on prefrontal cortical activity in humans, by exploiting known functional genetic variants that have demonstrable effects on cortical dopamine and acetylcholine signaling *in vivo*. Specifically, we investigated dorsolateral prefrontal cortex (DLPFC) activity during working memory in healthy subjects for interactions between single nucleotide polymorphisms (SNPs) in genes encoding the D2 receptor (*DRD2*, rs1076560) and the nicotinic receptor α5 (*CHRNA5*, rs16969968). *DRD2* is located on chromosome 11 and encodes two D2 isoforms, D2S (short) and D2L (long). D2L receptors mainly mediate post-synaptic signaling, while D2S receptors mainly serve as auto-receptors on pre-synaptic neurons [43], even though they are also found on post-synaptic neurons [44]. The minor allele (T) of *DRD2* rs1076560 (G/T), located within intron 6 of *DRD2*, is associated with reduced expression of D2S in prefrontal cortex and striatum, and with altered activity of the striato-thalamic-prefrontal pathway during working memory in healthy subjects [45] and patients with schizophrenia [46]. *DRD2* rs1076560 genotype also predicts putative steady-state striatal dopamine as assessed with SPECT and its correlation with prefrontal activity during performance of working memory, in that subjects carrying the T allele have reduced striatal D2 signaling and increased prefrontal activity during the 2-Back working memory task [47]. Recently, the T allele has been associated with risk for substance abuse related disorders including alcohol dependence [48], cocaine abuse [49] and opioid addiction [50]. Moreover, other *DRD2* variants have been associated with nicotine dependence [51], [52] and alcoholism [53], [54]. *CHRNA5*, encoding the α5 nicotinic accessory subunit, is located on chromosome 15. The rs16969968 SNP within *CHRNA5* changes the encoded amino acid sequence from aspartic acid (G allele) to asparagine (A allele) at position 398 (Asp398Asn) [55], [56]. The A allele resides almost exclusively on a haplotype associated with reduced *CHRNA5* mRNA expression in the brain [57], [58]. Furthermore, it has been associated *in vitro* with lower agonist-evoked intracellular calcium response of α4β2α5 nAChRs, lower Ca^2+^ permeability and greater short-term desensitization compared to the α5 ancestral allele (G) [55], [59]. Moreover, the A allele has also been associated with increased risk for lung cancer [55], nicotine dependence and smoking behavior [60], [61], as well as with lower cognitive performance in healthy subjects [62]. More recently, two studies have demonstrated that this allele is also associated with increased susceptibility to schizophrenia and bipolar disorders [63], [64].

Altogether, these findings suggest the crucial functional relevance of D2 and nAChR receptors as well as of genetic variation in *DRD2* and *CHRNA5* for prefrontal physiology. Furthermore, they implicate a complex and tight relationship between D2 and nAChR signaling, and call for further investigation of the impact of related genetic interaction on brain function [55]. Indeed, understanding the effect of genetic interactions on brain function has immediate clinical potential in elucidating the pathophysiology of complex neuropsychiatric disorders (i.e. Alzheimer Disease, Parkinson Disease, Schizophrenia) and in predicting therapeutic drug response [65].

Guided by the hypothesis that the DLPFC is especially vulnerable to the combined effect of suboptimal dopaminergic and cholinergic signaling, the aim of the present study was to investigate in healthy subjects the effect of *CHRNA5* rs16969968 and its interaction with *DRD2* rs1076560 on prefrontal physiology (as assessed with blood oxygenation level-dependent functional magnetic resonance imaging, BOLD fMRI), and mediated behavior during working memory. Furthermore, given compelling evidence in animals that both dopamine and acetylcholine signaling are involved in brain development and in ongoing local synaptic plasticity [37], [66], we also explored the potential effect of these two polymorphisms and their interaction on prefrontal gray matter volume.

## Materials and Methods

### Participants

Healthy Caucasian subjects from the region of Puglia, Italy, were recruited for the study and were evaluated with the Structured Clinical Interview for DSM-IV [67] to exclude any psychiatric disorder. Further exclusion criteria were: history of drug or alcohol abuse, active drug use in the past year, head trauma with loss of consciousness, and any significant medical condition revealed by clinical and magnetic resonance imaging. Handedness (Edinburgh Inventory)[68], and total IQ (WAIS-R) were also measured. The present study was approved by the local Institutional Review Board (Comitato Etico Locale Indipendente Azienda Ospedaliera “Ospedale Policlinico Consorziale” Bari). After complete description of the protocol and procedures, written informed consent was obtained by all participants, in accordance with the Helsinki Declaration. All subjects were genotyped for *CHRNA5* rs16969968 and *DRD2* rs1076560 and underwent one or more of the procedures described below.

The study involved a total number of 460 healthy subjects, with overlapping groups undergoing behavioral assessments, functional MRI (fMRI), and structural MRI (sMRI). A sample of 387 subjects (age, mean ± SD: 26.6±7.8; 194 males) underwent working memory behavioral assessment. A sample of 329 individuals (age: 27.1±7.8; 161 males) underwent fMRI during the N-Back working memory task, and a group of 211 individuals (age: 26.5±7.4; 114 males) underwent sMRI for Voxel Based Morphometry analysis. 166 subjects performed both fMRI and sMRI, 274 subjects performed both fMRI and WM behavioral assessment, while 173 subjects performed both sMRI and WM behavioral assessment.

In order to exclude that nicotine consumption may have been a confounding factor for our results, we also evaluated smoking status. Smokers were defined as those who smoked for at least 1 year and were currently smoking [69]. Chronic exposure was estimated in packs-year. All smokers were not allowed to tobacco use at least for 2 hours before scanning. Non smokers were defined by lifetime smoking of less than 20 cigarettes. Smoking status was available for a total of N = 221 subjects. More specifically, neuropsychological analyses were performed in a sample of N = 205 subjects, 114 Non-Smokers and 91 Smokers (age, mean ± SD: 26.42±6.90; 98 males). fMRI analyses were performed in a sample of N = 204 subjects, 137 Non-Smokers and 67 Smokers (age, mean ± SD: 26.42±6.90; 93 males).

#### Genotype determination

DNA was extracted from whole blood samples using standard procedures. *CHRNA5* rs16969968 genotypes were determined by restriction fragment length polymorphism methods, using primers tagged with a fluorophore (forward 5′-TAGAAACACATTGGAAGCTGCG-3′ and reverse 5′- AATTCTGGCCCTCAATCTATGCT-3′). Taqα1 (from New England Biolabs, Ipswich, MA, USA) was used to cut the amplified gDNA ancestral allele, and the resultant fragment length was resolved and analyzed on an ABI 3730 DNA analyzer (Life Technologies). *DRD2* rs1076560 genotypes were determined by direct sequencing. Amplification of the 213 bp DNA fragment containing the *DRD2* rs1076560 polymorphism (G>T) was performed using forward 5′-GGCAGAACAGAAGTGGGGTA-3′ and reverse 5′-GACAAGTTCCCAGGCATCAG-3′ primers. PCR was performed on 100 ng genomic DNA in a standard 25 µL volume, containing 0.2 µM primers, 100 µM dNTPs, 2.5 µl reaction Gold buffer (Applied Biosystems, Foster City, CA), 2 mM MgCl2 and 2.5 U Ampli Taq Gold Polymerase (Applied Biosystems, Foster City, CA). Thermal cycler conditions were as follows: initial denaturation step at 94°C for 12 min; 94°C for 30 sec, 62°C for 30 sec, 72°C for 45 sec for 35 cycles; final elongation step at 72°C for 7 minutes. *DRD2* rs1076560 PCR products were sequenced in both directions using BigDye Terminator chemistry and run on an ABI Prism 3130 DNA sequencer (Applied Biosystems, Foster City, CA, USA). Sequences were analyzed with SeqMan from Lasergene-DNASTAR package (DNASTAR Inc., Madison, Wis.).

All alleles displayed Hardy-Weinberg equilibrium. Given the low number of subjects homozygous for the *DRD2*-T minor allele, we combined these individuals (when present) with heterozygous subjects (GT) for further analyses, consistent with earlier studies evaluating polymorphisms with low minor allele frequencies [70]. In each of the study cohorts included in the experiments, the χ^2^ analysis demonstrated equal distribution of *DRD2* genotypes in *CHRNA5* groups and vice versa (all χ^2^<4.06; all p>0.13), indicating that the genotype groups were not differentially distributed in subpopulations.

#### N-Back Working Memory paradigm for behavioral study

Briefly, ‘N-back’ refers to how far back in the sequence of stimuli the subject had to recall. The stimuli consisted of numbers (1–4) shown in random sequence and displayed at the points of a diamond-shaped box. There was a visually paced motor task which also served as a non-memory guided control condition (0-Back) that simply required subjects to identify the stimulus currently seen. In the working memory conditions, the task required recollection of a stimulus seen one (1-Back) or two stimuli (2-Back) previously while continuing to encode additionally incoming stimuli. Performance data were recorded as the percentage (%) of correct responses (accuracy) and as reaction time (ms).

#### Statistical Analysis for demographics and behavioral performance

One-way ANOVAs and χ^2^ analyses were used to compare demographic data across genotype groups. General linear models with repeated measures for task conditions (1-Back and 2-Back) and with predictors *CHRNA5* rs16969968 and *DRD2* rs1076560 were used to evaluate behavioral differences across genotype groups. Fisher's Least Significant Difference Test and t-tests for dependent samples as appropriate were used for all *post-hoc* analyses.

### Imaging Data Acquisition and Processing

Functional and structural MRI were performed on a General Electric (Milwaukee, WI) 3 Tesla scanner.

#### fMRI acquisition parameters

Each subject was scanned using a gradient-echo echo planar imaging sequence (repetition time, 2000 ms; echo time, 28 ms; 20 interleaved axial slices; thickness, 4 mm; gap, 1 mm; voxel size, 3.75×3.75×3.75; flip angle, 90°; field of view, 24 cm; matrix, 64×64). We used a simple block design in which each block consisted of eight alternating 0-Back and 2-Back conditions (each lasting 30 s), obtained in 4 min and 8 s, 120 whole-brain fMRI volumes. The first four scans at the beginning of each time series were acquired to allow the signal to reach a steady state and were not included in the final analysis.

#### fMRI image analysis. Preprocessing and statistical analyses

Data processing and analysis were performed with freely available Statistical Parametric Mapping software (SPM8; Wellcome Trust Centre for Neuroimaging, London, UK, http://www.fil.ion.ucl.ac.uk/spm). Images, for each subject, were realigned to the first volume in the time series and movement parameters were extracted to exclude subjects with excessive head motion (>2 mm of translation, >2° rotation). Images were then re-sampled to a 2 mm isotropic voxel size, spatially normalized into a standard stereotactic space (Montreal Institute on Neurology, MNI template) and smoothed using a 10 mm full-width half-maximum isotropic Gaussian kernel to minimize noise and to account for residual inter-subject differences. A box car model convolved with the hemodynamic response function at each voxel was modeled. In the first-level analysis, linear contrasts were computed producing a t statistical map at each voxel for the 2-Back condition, assuming the 0-Back condition as a baseline.

All the individual contrast images were entered in a second level random effects analysis. A Factorial Analysis of Variance (ANOVA) was then performed, with *CHRNA5* rs16969968 and *DRD2* rs1076560 genotype as the between-subjects factors. Because of our strong hypothesis about α4β2α5 nAChRs and dopamine-D2 mediated modulation of dorsolateral prefrontal neuronal plasticity, we used a statistical threshold of p<0.05, with family-wise error (FWE) small-volume correction within a Region of Interest (ROI) comprehensive of Brodmann's areas 46 (BA46) as defined by the Wake Forest University PickAtlas 1.04 (WFU_PickAtlas) (http://www.fmri.wfubmc.edu/cms/software#PickAtlas). Because we did not have *a priori* hypotheses regarding the activity of brain regions outside of the ROI we used a statistical threshold of p<0.05, FWE-corrected for these whole-brain comparisons. Because no effects were detected with this threshold and for the sake of completeness, we also report exploratory analyses at p = 0.001 uncorrected, k = 10. Moreover, to further explore differences between genotype groups, *post-hoc* analysis outside of SPM8 was also performed on BOLD responses extracted from the cluster showing the interaction using MarsBar (http://marsbar.sourceforge.net/).

Finally, to evaluate the behavioral relevance of the interaction between *CHRNA5* rs16969968 and *DRD2* rs1076560 genotypes on DLPFC activity, we performed separate linear regression analyses within SPM8 using as predictor behavioral accuracy (%) at 2-Back working memory task both in the whole sample and within each genotype group. Again, a statistical threshold of p<0.05, with FWE small-volume correction within a ROI comprehensive of BA46 as defined by the WFU_PickAtlas was applied. All fMRI data are reported with reference to the MNI standard space within SPM8.

#### sMRI acquisition parameters

Three-dimensional images were acquired using a T1-weighted SPGR sequence (TE = min full; flip angle, 6°; field of view, 250 mm; bandwidth, 31.25; matrix, 256×256) with 124 1.3-mm-axial slices.

#### sMRI image analysis. Preprocessing and statistical analyses

Voxel Brain Morphometry Analysis (VBM) of the sMRI data was also performed using SPM8. The T1-weighted scans were partitioned into different tissue classes- gray matter (GM), white matter and non-brain voxels (cerebrospinal fluid, skull) - based on separate tissue probability maps for each tissue class using the “new segmentation” approach in SPM8 [71]. In order to compare brains of different subjects, the resulting segments were normalized to a population template generated from the complete dataset using a diffeomorphic registration algorithm [72]. This high-dimensional non-linear warping algorithm selects conserved features, which are informative for registration, thus minimizing structural variation among subjects and providing optimal inter-subject registration. Subsequently, all images were “modulated” by the Jacobian determinants from the normalisation steps to preserve initial volumes. Thus, images were smoothed by convolution with an isotropic Gaussian kernel of 8 mm full-width at half maximum.

We examined the SNP main effects and their interaction by creating voxel-based, whole-brain, statistical parametric maps using Gaussian random fields theory and the general linear model. More specifically, we used a full factorial Analisys of Covariance (ANCOVA) design with two level factors, *DRD2* rs1076560 and *CHRNA5* rs16969968. The statistical model also included orthogonalized first- and second-order polynomials of age, gender and total GM volume as “nuisance” variables, in order to control for any independent effects on our findings and to ensure that the analysis identified regionally specific “non-global” effects [73]. Because of our strong a priori hypothesis based on the effects of *CHRNA5* and *DRD2* variants on mRNA levels in prefrontal cortex [45], [57] and consistent with the fMRI analyses, the ANCOVA was masked with an ROI identified in BA46 using the WFU_PickAtlas. Statistical non-stationary inference [74] was performed at the cluster level at p<0.05 corrected within the ROI by using the ns toolbox (http://fmri.wfubmc.edu/cms/NS-General) implemented in SPM8, to avoid increased false-positive rate due to the non-stationary structural images. Exploratory whole-brain statistics outside the ROI was set at p = 0.001, uncorrected.

VBM results are reported with reference to the MNI standard space within SPM8. To further examine differences between genotype groups, *post-hoc* analysis outside of SPM8 was also performed on gray matter volumes extracted from the cluster showing a *CHRNA5* rs16969968 by *DRD2* rs1076560 interaction using MarsBar.

## Results

Demographics (±SD) and genetics of the samples included in the experiments are reported in Table 1.

**Table 1:** Demographics (±SD) and genetics of the samples included in the experiments performed.

	Cognitive Behavior	fMRI	sMRI
**N**	387	329	211
Gender (M/F)	194/193	161/168	114/97
Age	26.61±7.76	27.06±7.76	26.47±7.42
Handedness	0.73±0.42	0.77±0.36	0.62±0.51
IQ	107.57±12.48	109.81±12.38	107.77±12.62
**N**			
*CHRNA5* GG/*DRD2* GG	122	99	72
*CHRNA5* GG/*DRD2* Tcarriers	28	21	15
*CHRNA5* GA/*DRD2* GG	143	117	76
*CHRNA5* GA/*DRD2* Tcarriers	42	45	23
*CHRNA5* AA/*DRD2* GG	42	36	28
*CHRNA5* AA/*DRD2* Tcarriers	10	11	7

### Association with Working Memory behavioral performance

**Figure 1:**
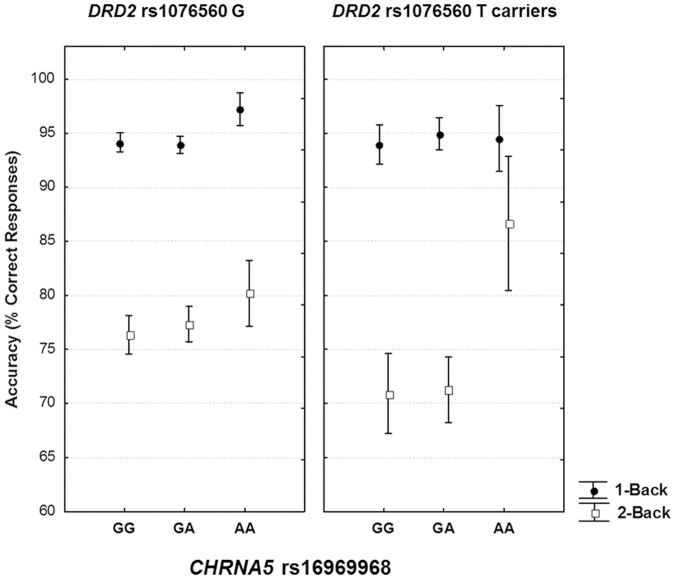
Interaction between Working Memory behavioral performance, *DRD2* rs1076560 and *CHNRA5* rs16969968 genotypes. Mean ± Standard Errors correct responses (*DRD2*-GG, left panel; *DRD2*-Tcarriers, right panel) showing the interaction between the two genotypes. See text for statistics.

In the cognitive behavior sample (N = 387), genotype groups were matched in terms of gender, age, handedness, and IQ (all p>0.1). Repeated measures ANOVA on working memory load accuracy indicated no significant effect of *DRD2* rs1076560 (F_1,381_ = 0.18, p = 0.66); a main effect of *CHRNA5* rs16969968 (F_2,381_ = 3.10, p = 0.046) and an interaction between *DRD2* rs1076560 and *CHRNA5* rs16969968 (F_2,381_ = 3.16, p = 0.044) [Mean Squared Error (MSE): 1-Back = 94, 2-Back = 384] (Fig. 1). More specifically, *post hoc* analysis with t-test for dependent samples demonstrated a statistically significant drop in performance from 1-Back to 2-Back for all genotype groups (all p<0.001) with the exception of *CHRNA5* AA/*DRD2* GT subjects (p = 0.09) (Fig. 1). In other words, the interaction between the minor T allele of rs1076560 and the minor A allele of rs16969968 was associated with attenuated drop in performance which was instead observed from 1-Back to 2-Back for all other genotypes. Repeated measures ANOVA on working memory load reaction time indicated no significant effect of *DRD2* rs1076560 (F_1,381_ = 3.66, p = 0.07); no significant effect of *CHRNA5* rs16969968 (F_2,381_ = 1.04, p = 0.35), and no interaction between *DRD2* rs1076560 and *CHRNA5* rs16969968 (F_2,381_ = 1.18, p = 0.307).

To test whether nicotine consumption may have confounded these results, we also performed ANCOVA covarying for smoking status in N = 205 subjects, including 114 Non-Smokers and 91 Smokers. Similar to the above analysis, ANCOVA demonstrated an interaction between *DRD2* rs1076560 and *CHRNA5* rs16969968 on working memory load accuracy (F_2,198_ = 5.10; p = 0.007; MSE 1-Back = 77.1, 2-Back = 327.4). More specifically, *post hoc* analysis with t-test for dependent samples demonstrated a statistically significant drop in performance from 1-Back to 2-Back for all genotype groups (all p<0.0001) with the exception of *CHRNA5* AA/*DRD2* GT subjects (p = 0.25). No significant genotype effects or interactions on working memory load reaction time were detected in this sample (all p>0.07).

### Association with Working Memory DLPFC activity measured with fMRI

In the fMRI sample (N = 329), genotype groups were also matched in terms of gender, age, handedness and IQ (all p>0.1). No genotype effects or interaction were present on accuracy and reaction time at the N-Back task in this sample (all p>0.1), thus allowing us to compare brain responses in the absence of behavioral differences. For behavioral performance see Table S1.

#### Effect of the Working Memory task

As expected from previous studies with the N-Back task (Callicott et al.1999, 2000; Bertolino et al. 2004, 2006), performance of the 2-Back working memory condition was associated with activity in a distributed network of brain regions including the prefrontal cortex, the parietal cortex, the anterior cingulate, and the striatum bilaterally.

#### Genotype main effects and interaction during working memory

**Figure 2:**
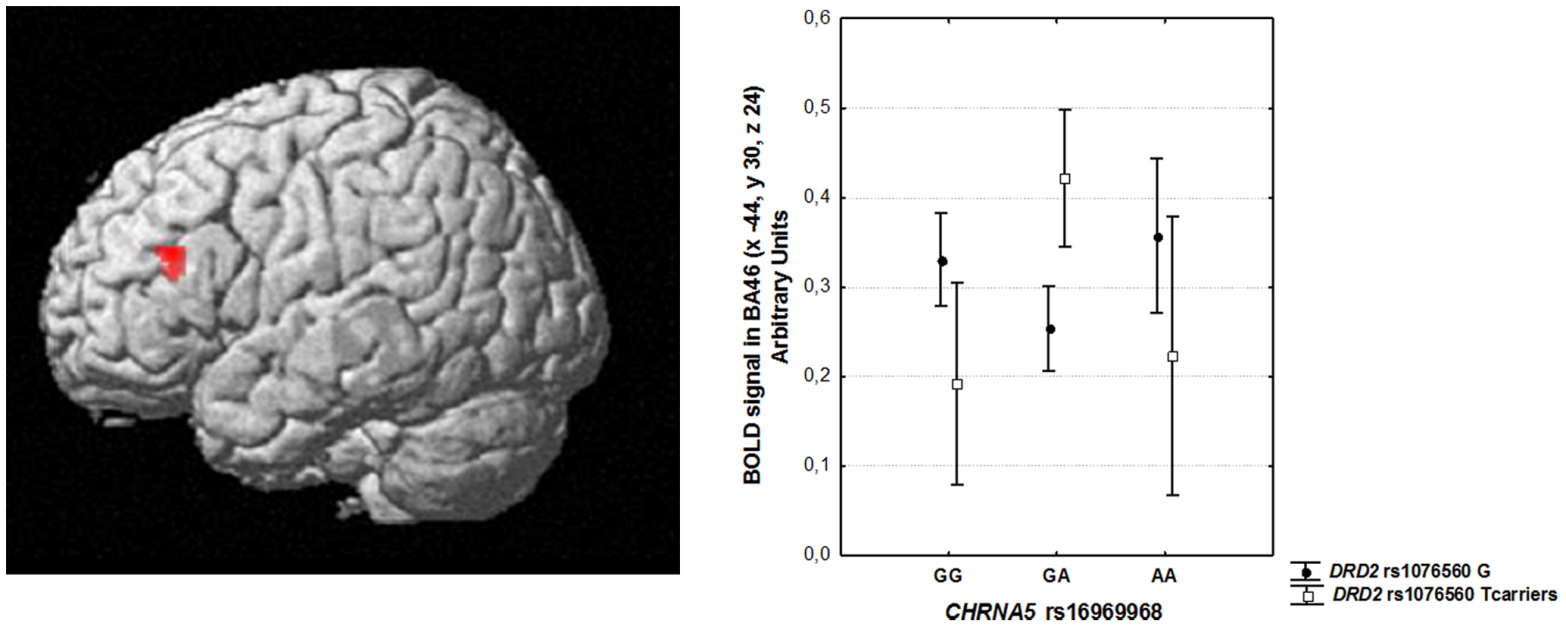
Interaction between *DRD2* rs1076560 and *CHNRA5* rs16969968 on prefrontal physiology at 2-Back. 3Dimensional rendering (left, image thresholded at p<0.005, uncorrected) and mean ±0.95 CIs of BOLD response (right) of the interaction between *DRD2* rs1076560 and *CHNRA5* rs16969968 on working memory DLPFC activity.

No statistically significant main effect of *CHRNA5* rs16969968 or *DRD2* rs1076560 genotype in the DLPFC ROI was found. On the other hand, ANOVA revealed a *DRD2* by *CHRNA5* interaction in left DLPFC (BA 46: x -44 y 30 z 24; K = 28; corrected pFWE = 0.036; Fig. 2). *Post hoc* analysis of BOLD response from this cluster indicated that within *DRD2*-GT genotype, *CHRNA5*-GA subjects have greater prefrontal activity compared with *DRD2*-GT *CHRNA5*- GG (p = 0.02) or -AA subjects (p = 0.02) (Fig. 2b). No significant differences emerged in the context of *DRD2*-GG genotype. Results of the uncorrected exploratory whole-brain analyses are reported in Table 2.

**Table 2:** Results of exploratory uncorrected whole brain statistics (p<0.001, k = 10) showing the main effect of *DRD2* and its interaction with *CHRNA5* on brain physiology at 2-Back WM Task.

Main effect of *DRD2* genotype							
						MNI	
Region	Brodmann Area	*k*	Z-score	*p*	x	y	z
Middle Temporal Gyrus	BA 39	19	3.59	0.0001	40	−54	22
Middle Frontal Gyrus	BA 10	15	3.35	0.0001	32	56	6

Interaction of *CHRNA5* and *DRD2* genotypes							
						MNI	
Region	Brodmann Area	*K*	Z-score	*P*	x	y	z
Inferior Parietal Lobe	BA 40	98	4.17	0.0001	−36	−46	50
Middle Frontal Gyrus	BA 10	29	3.58	0.0001	30	52	0
Middle Frontal Gyrus	BA 46	23	3.58	0.0001	−46	30	28
Middle Frontal Gyrus	BA 6	21	3.51	0.0001	−20	10	64

Again, to test whether nicotine consumption may have confounded these results, we also performed ANCOVA covarying for smoking status on BOLD responses identified in the above analysis. This analysis included N = 204 subjects, of whom 137 were Non-Smokers and 67 Smokers. These analysis indeed demonstrated an interaction between *DRD2* by *CHRNA5* (p = 0.008). Similar to the analysis in the whole sample, *post hoc* analysis indicated within *DRD2*-GT genotype, *CHRNA5*-GA subjects have greater prefrontal activity compared with *DRD2*-GT *CHRNA5*- GG (p = 0.01) or -AA subjects (p = 0.007). No significant differences emerged in the context of *DRD2*-GG genotype. These results suggest that smoking status did not significantly confound the identified interaction.

#### Relationship between DLPFC activity and behavioral performance at 2-Back

**Figure 3:**
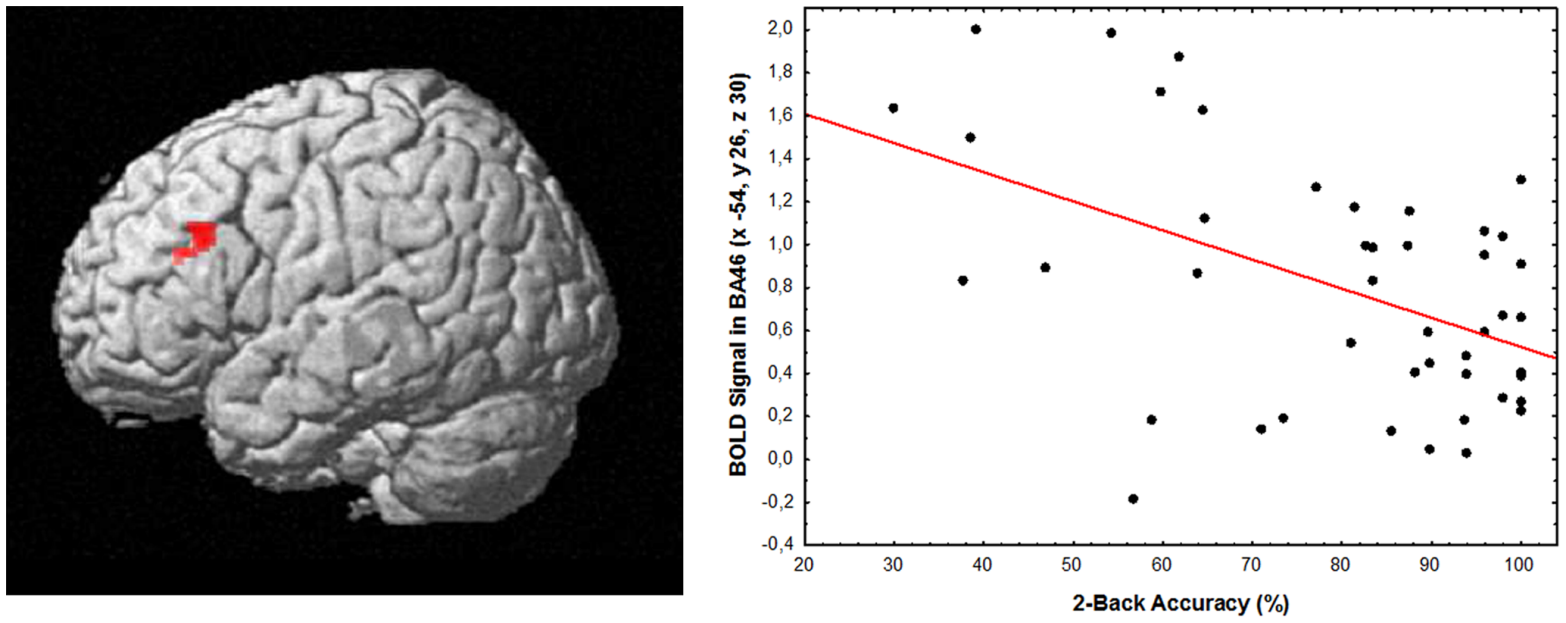
Correlation between BOLD fMRI in prefrontal cortex and Working Memory accuracy in *CHRNA5*-GA/*DRD2*-GT subjects. 3Dimensional rendering (left, image thresholded at p<0.005, uncorrected) of the correlation between BOLD response in DLPFC and percentage of correct responses at 2-Back task in *CHRNA5*-GA/*DRD2*-GT subjects. On the right, relative scatterplot is shown indicating individual data points.

Regression analysis in SPM8 demonstrated a negative correlation between activity in DLPFC and accuracy (%) at 2-Back in the *CHRNA5*-GA/*DRD2*-GT group (BA 46: x -56 y 26 z 30; K = 39; corrected pFWE = 0.02; Fig. 3). Also, exploratory analyses which did not survive correction for multiple comparisons suggested a negative correlation in the *CHRNA5*-AA/*DRD2*-GT group (x -42 y 30 z 22; K = 15; p = 0.002 uncorrected), and a positive correlation in the *CHRNA5*-GG/*DRD2*-GT group (x -36 y 56 z 30; K = 20; p = 0.002 uncorrected) (See Fig. S1).

### Association with DLPFC gray matter volume measured with sMRI

In the sMRI sample (211 subjects), genotype groups were also matched in terms of gender, age, handedness and IQ (all p>0.1).

#### Genotype main effects and interaction

**Figure 4:**
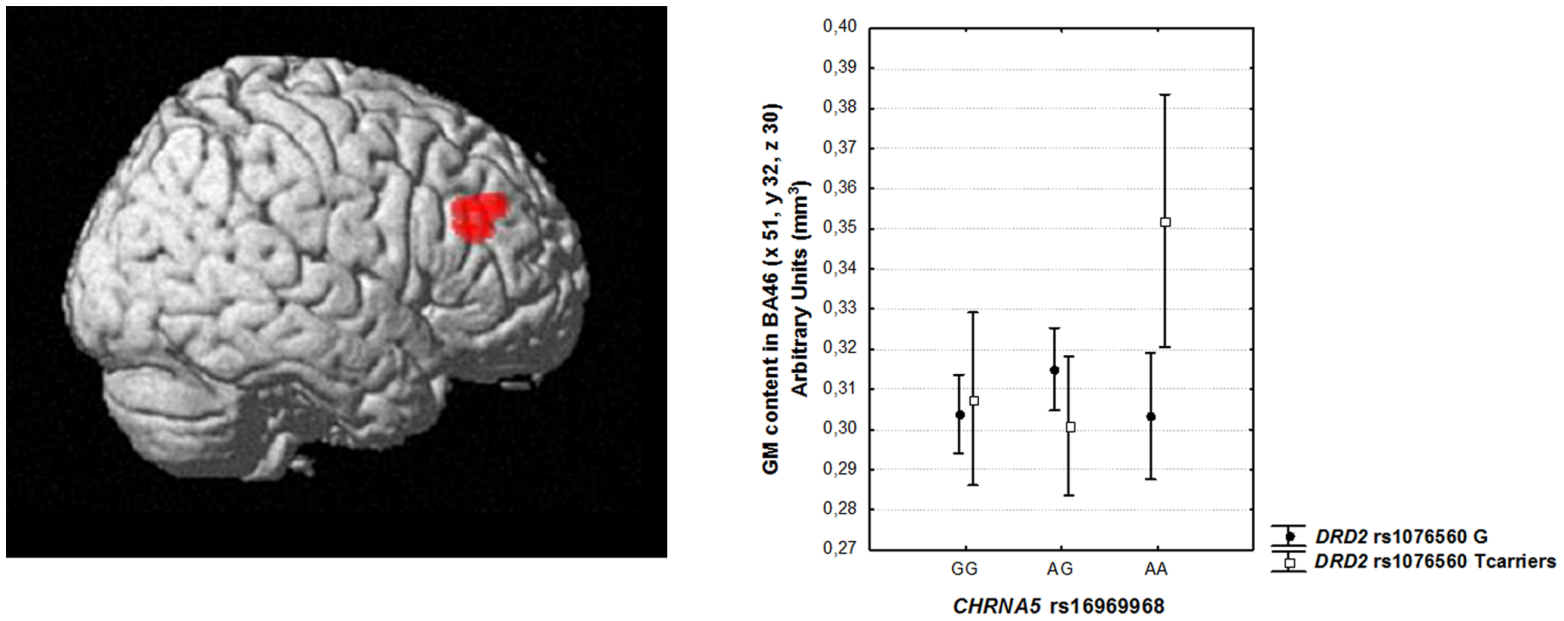
Interaction between *DRD2* rs1076560 and *CHNRA5* rs16969968 on prefrontal gray matter volume. 3Dimensional rendering (left, image thresholded at p<0.005, uncorrected) and mean ±0.95 CIs of gray matter content (right) of the interaction between *DRD2* rs1076560 and *CHNRA5* rs16969968 on DLPFC gray matter.

There was no statistically significant main effect of *CHRNA5* rs16969968 or of *DRD2* rs1076560 genotype in the DLPFC ROI. However, an interaction between *CHRNA5* and *DRD2* genotypes was found in right DLPFC (BA 46: x 51, y 32, z 30, k = 321, Z = 3.96, p = 0.006 cluster-level corrected; Fig. 4). *Post hoc* analysis of gray matter volume extracted from the interaction cluster indicated that *CHRNA5*-AA/*DRD2*-GT subjects have greater DLPFC gray matter volume compared to all other genotype groups (all p<0.02; Fig. 4).

Results of the uncorrected exploratory whole-brain analyses are reported in Table 3.

**Table 3:** Results of exploratory uncorrected whole brain statistics (p<0.001, k = 10) showing the main effect of genotypes and their interaction on gray matter volume.

Main effect of *CHRNA5* genotype							
						MNI	
Region	Brodmann Area	*K*	Z-score	*p*	x	y	z
Inferior Frontal Gyrus	BA 47	169	4.01	0.0001	−28	30	−5
Occipital Lobe, Cuneus	BA 19	137	3.82	0.0001	−10	−95	30
Cerebellum, Posterior Lobe		466	3.68	0.0001	28	−82	−24

Main effect of *DRD2* genotype							
						MNI	
Region	Brodmann Area	*K*	Z-score	*p*	x	y	z
Occipital Lobe	BA 17	43	3.77	0.0001	−20	−60	−3
Parahippocampal Gyrus		52	3.55	0.0001	34	−1	−26
Amygdala							
Middle Occipital Gyrus	BA 18	15	3.41	0.0001	26	−87	−5
Posterior Cingulate	BA 31	132	3.33	0.0001	26	−61	19
Parahippocampal Gyrus		51	3.32	0.0001	−21	−7	−15
Amygdala							
Cerebellum, Cerebellar Tonsil		28	3.20	0.0001	27	−58	−60

Interaction of *CHRNA5* and *DRD2* genotypes							
						MNI	
Region	Brodmann Area	*K*	Z-score	*P*	x	y	z
Middle Frontal Gyrus	BA 46	312	4.01	0.0001	51	32	28
	BA 10	206	3.77	0.0001	12	63	4
Parietal Lobe	BA 7	87	3.58	0.0001	22	−49	56
Middle Frontal Gyrus	BA 10	12	3.56	0.0001	26	63	25
	BA 47	13	3.54	0.0001	42	36	−3
Cingulate Gyrus	BA 24	33	3.46	0.0001	8	−13	37
Cerebellum, Declive		136	3.31	0.0001	22	−85	−30

## Discussion

The results of the present study demonstrate that variants in two genes implicated in dopamine and acetylcholine signaling interact to modulate the biology and physiology of the prefrontal cortex during working memory. More specifically, the interaction between *CHNRA5* rs16969968 and *DRD2* rs1076560 genotypes differentially predicted cognitive behavior with increasing working memory load, in that *CHRNA5*-AA/*DRD2*-GT subjects have better behavioral performance. In addition, we found that the effect of *CHRNA5* rs16969968 in dorsolateral prefrontal activity at 2-Back is only evident in the context of *DRD2* rs1076560 genotype, such that *CHRNA5* demonstrates an inverted U shaped prefrontal response in *DRD2*-GT subjects (see below).

As a further demonstration of the functional effects of these polymorphisms, *CHRNA5* rs16969968 and *DRD2 rs1076560* also interacted on gray matter volume of the dorsolateral prefrontal cortex (DLPFC). Once again, the effect of *CHNRA5* was mostly evident in the context of *DRD2*-GT genotype.

Our behavioral findings during working memory as elicited by the N-Back task are consistent with a previous report by Markett et al. (2010) in healthy subjects (N = 101), showing an interaction between a functional SNP in *CHRNA4* (rs1044396) and a haplotype block covering three SNPs in *DRD2* (rs1800497, rs6277, rs2283265) on working memory capacity [75]. As in our sample, this effect only became apparent at greater working memory load, suggesting that the *CHRNA5* by *DRD2* interaction affects the efficiency by which relevant information is encoded during the trial-wise updating of working memory items. Unlike all other genotype groups, *CHRNA5*-AA/*DRD2*-GT subjects showed no statistically significant reduction in behavioral performance with increasing working memory load, leading to the speculation that the prefrontal neuronal *signal-to-noise* affected by the genetically determined balance of cholinergic and dopaminergic signaling is increased in CHRNA5-AA/DRD2-GT subjects allowing greater performance with increasing working memory load. Moreover, this interaction is similar to the reciprocal rescue of minor allele risk found in association with other biological phenotypes [76]. Of note, our results suggest that the pattern of genotype effect on working memory load accuracy was not moderated by reaction time.

Our functional imaging data also indicate that the interaction between *CHRNA5* rs16969968 and *DRD2* rs1076560 *g*enotypes differentially predicted the efficiency of the prefrontal cortex at 2- Back condition. Earlier fMRI studies found that genetic variation in dopamine signaling in the prefrontal cortex affects the efficiency or *signal-to-noise* ratio of the physiological response during the N-Back following an inverted U-shaped response function [77]–[79]. In the present study, we found that only within *DRD2*- GT genotype, *CHRNA5* demonstrates an inverted U shaped prefrontal response at 2-Back working memory task, suggesting that *CHRNA5* rs16969968 further affects the *signal-to-noise ratio* of prefrontal cortex in subjects with greater dopamine signaling (i.e. GT subjects) [45]–[47]. More specifically, within the context of *DRD2*-GT genotype: *CHRNA5*-AA subjects are more efficient, because the combination of behavioral data and imaging results suggests that they have reduced activity for greater behavioral performance; *CHRNA5*-AG subjects are less efficient, because they show greater activity for reduced behavioral performance, which is also consistent with the negative relationship between BOLD response and behavioral accuracy; and *CHRNA5*-GG have reduced engagement of prefrontal resources to the task at hand for reduced behavioral performance, as also suggested by the positive relationship between prefrontal activity and behavioral performance.

Thus, the *DRD2* GT subjects show an overdominance effect for the *CHRNA5* genotype with heterozygous revealing greater/more inefficient prefrontal activity compared with individuals homozygous for either allele. While this effect in genetics would be regarded as a “heterozygous advantage”, at the brain imaging level it may actually reflect an inefficient prefrontal activity during information processing. This finding is of particular interest since central dopamine-acetylcholine imbalance in synaptic plasticity is responsible for cognitive deficits in Parkinson disease [80] and likely in psychosis, as suggested by heavy smoking in patients with schizophrenia [81].

There may be several and complex molecular/neuronal mechanisms in human DLPFC which are responsible for the interaction we have measured *in vivo* with BOLD fMRI, and further work on molecular and cellular models is warranted. Still, previous work examining cortical anatomy and physiology allows us to speculate on the biology underlying our observations. D2 receptors in prefrontal cortex are mainly found pre-synaptically on dopamine terminals [82], modulating dopamine release and D2S autoreceptors are relatively more abundant in the prefrontal cortex compared to D2Ls [45]. Thus, *DRD2*-GT genotype associated with reduced D2S may increase dopamine levels in the prefrontal cortex, and in turn increase its activity. nAchRs containing the α5 subunit in prefrontal cortex are mainly expressed on soma and axon of LVI pyramidal neurons [29], [83], where they are responsible for strong activation of the neuronal population of this layer [29]. Similarly to *DRD2* genotype, the *CHRNA5*-A allele which is associated with reduced total *CHRNA5* mRNA expression in prefrontal cortex tissue and signaling *in vitro*, may alter the neuronal activation of LVI pyramidal neurons. However, as mentioned above, the effect of *CHRNA5* rs16969968 is only manifest in the context of *DRD2* rs1076560 GT genotype, suggesting that the physiological relevance of this *SNP*, in terms of overall prefrontal cortex activity during working memory, occurs only in the context of genetic variation modulating dopamine signaling.

Alternatively, the interaction observed in prefrontal cortex could also be influenced by activity within the cortico-striato-thalamic pathway. Dopamine is an important modulator of this circuit. Specifically, greater release of dopamine in the striatum increases activity of the whole network. We have previously demonstrated in healthy subjects that *DRD2*-GT genotype is associated with reduced pre-synaptic DAT and post-synaptic D2 receptor density, reduced striatal dopamine signaling [47], greater caudate activity and greater prefrontal activity during working memory performance [46]. The α4β2α5 nAChRs in striatum are expressed on dopaminergic terminals [84], where they dominantly regulate dopamine release in the dorsal caudate-putamen [85], overriding the release mediated by ascending dopaminergic somata firing [86]. Thus, it is possible that the *DRD2* by *CHRNA5* genotype interaction might be associated with modulation of dopamine release in the striatum, which would increase activity in the whole circuit. However, we did not detect any *CHRNA5* rs16969968 effect or interaction with *DRD2* rs1076560 on striatal activity, and further studies are necessary to test this hypothesis.

The behavioral and functional imaging findings complement our VBM results, which provide *in vivo* evidence that *DRD2* and *CHRNA5* interact to affect gray matter volume in human DLPFC. This is consistent with previous data in animal models demonstrating an effect of dopamine and acetylcholine in brain development and in ongoing local neural plasticity [66], [87]. Increasing synaptic dopamine in developing brains through prenatal cocaine exposure leads to specific neurodevelopmental alterations including abnormal dendritic growth and abnormal arborization of pyramidal cells that persist postnatally [88]. Conversely, neonatal dopamine denervation in rat produces permanent differential changes in prefrontal cortex dendritic morphology, i.e. atrophy of proximal apical and basilar dendrites [89]. Cholinergic inputs to the cortex also appear early during brain development and are widespread in rat by the third week of post-natal life [87], [90], likely influencing the normal morphological development of pyramidal neurons. Two elegant studies have indicated that direct nicotinic stimulation can modulate growth or retraction of neurites in cultured neurons [91], [92]. More recently, Bailey et al (2012) have demonstrated that α5 nAChRs underlie the neurodevelopmental peak in the nicotinic excitation of murine medial prefrontal cortex LVI neurons that occurs during the third week of postnatal life, and that it is likely to influence a specific ontogenetic retraction of apical dendrites in LVI pyramidal neurons. However, it is possible that α5 nAChRs on dopaminergic neurons [33], [34] may also influence the morphology of prefrontal cortex neurons.

Some potential limitations of the present study should be addressed. First, the study includes assumptions that we expect protein expression to correspond to mRNA levels, as we did not directly measure dopamine and acetylcholine receptor levels in our cohort, we can only speculate about the molecular mechanisms relying on previous studies demonstrating functional consequences of the chosen SNPs. Hence, it remains to be determined whether and how genetic variation in dopaminergic and cholinergic signaling to cortical *signal-to-noise* may directly affect differential engagement of DLPFC at a cellular level, especially in humans. Second, our neuropsychological findings are based on a relatively small group size for the *CHRNA5* AA/*DRD2* GT genotype (N = 10). Although computation of the maximal effect size *d* and achieved power (respectively, 1.1 and 0.82) support their statistical robustness, replication of our results is necessary. Furthermore, Levene's test indicates homogeneity of variance of behavioral performance (difference between 2-Back and 1-Back accuracy) across *DRD2/CHRNA5* genotype groups (MS Effect  = 106.33; MS Error  = 85.30; F = 1.24; p = 0.28) supporting that our results were not influenced by unequal population variances. Another caveat of our study is that our neuropsychological and fMRI findings could be affected by tobacco use of subjects. Rs16969968 has been associated with nicotine dependence [55], although it does not alter *per se* sensitivity of α4β2α5 nAChRs to nicotine [59]. However, the additional analyses we performed including smoking status as covariate allowed us to exclude it as confounding factor.

The present study advances our understanding of the *in vivo* interactions between dopamine and acetylcholine signaling in the prefrontal cortex, specifically through the *DRD2* and *CHRNA5* receptors. Our observations of these gene-gene interactions on neurophysiology and cognition begin to build a more solid foundation for explaining the neurobiology underlying complex human behaviors and lend insight into disease susceptibility. Furthermore, our results have relevant potential implications for the therapeutic approach of various neurological and psychiatric disorders in which altered cholinergic transmission potentially contributes to cognitive deficits, such as those observed in schizophrenia.

## Supporting Information

Figure S1 **Correlation between BOLD fMRI in prefrontal cortex and Working Memory accuracy in CHRNA5-AA/DRD2-GT subjects (S1a) and in CHRNA5-GG/DRD2-GT subjects (S1b).**
(DOCX)

Table S1 **Behavioral data (mean ± SD) at the 2-Back task for each genotype group.**
(DOCX)
