# Supplementary material for: DRD2/CHRNA5 Interaction on Prefrontal Biology and Physiology during Working Memory
Source: PLoS One. 2014 May 12;9(5):e95997. doi: 10.1371/journal.pone.0095997 (PMC4018353; doi:10.1371/journal.pone.0095997)

**Figure S1: Correlation between BOLD fMRI in prefrontal cortex and Working Memory accuracy in CHRNA5-AA/DRD2-GT subjects (S1a) and in CHRNA5-GG/DRD2-GT subjects (S1b).**


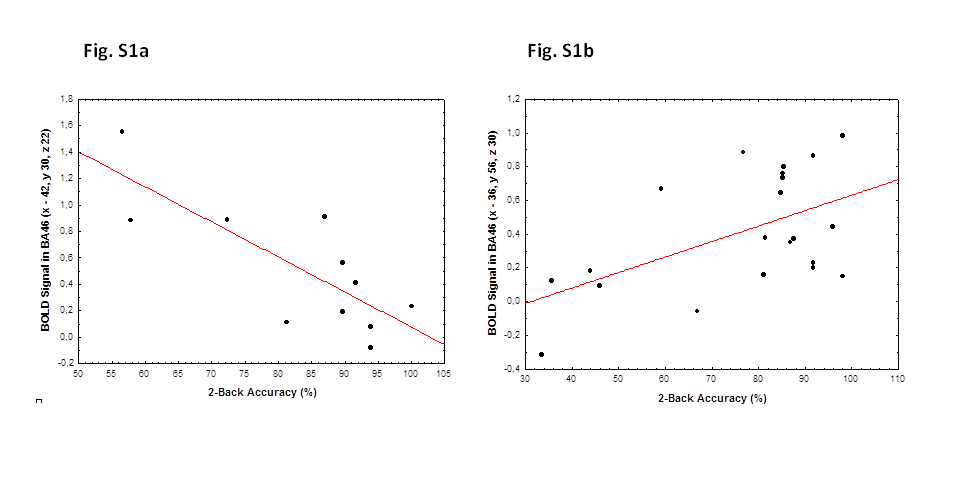

Supplement: Figure S1 — Correlation between BOLD fMRI in prefrontal cortex and Working Memory accuracy in CHRNA5-AA/DRD2-GT subjects (S1a) and in CHRNA5-GG/DRD2-GT subjects (S1b). (DOCX) [file pone.0095997.s001.docx]
