# Supplementary material for: DRD2/CHRNA5 Interaction on Prefrontal Biology and Physiology during Working Memory
Source: PLoS One. 2014 May 12;9(5):e95997. doi: 10.1371/journal.pone.0095997 (PMC4018353; doi:10.1371/journal.pone.0095997)
Supplement: Table S1 — Behavioral data (mean ± SD) at the 2-Back task for each genotype group. (DOCX) [file pone.0095997.s002.docx]

**Table S1: Behavioral data (mean ±SD) at the 2-Back task for each genotype group**

| ***CHRNA5_DRD2*** | **% Correct responses** | **Reaction time (ms)** |
| --- | --- | --- |
| **GG_GG** | 80.73±15.27 | 494.91±242.19 |
| **GG_Tcarriers** | 76.4±20.60 | 455.64±191.75 |
| **GA_GG** | 82.71±19.81 | 492.59±239.71 |
| **GA_Tcarriers** | 79.56±20.17 | 564.16±282.72 |
| **AA_GG** | 84.05±17.72 | 466.70±236.85 |
| **AA_Tcarriers** | 83.01±14.67 | 405.08±227.07 |
